# Supplementary material for: Natural Products Lysobactin and Sorangicin A Show In Vitro Activity against Mycobacterium abscessus Complex
Source: Microbiol Spectr. 2022 Oct 31;10(6):e02672-22. doi: 10.1128/spectrum.02672-22 (PMC9769517; doi:10.1128/spectrum.02672-22)
Supplement: Supplemental file 1 — Tables S1 to S4. Download spectrum.02672-22-s0001.pdf, PDF file, 0.3 MB [file spectrum.02672-22-s0001.pdf]

Natural products lysobactin and sorangicin A show *in vitro* activity against *Mycobacterium abscessus* complex

Jaryd R. Sullivan[1,2,3], Jacqueline Yao[2], Christophe Courtine[1,2], Andréanne Lupien[2,3], Jennifer Herrmann[4], Rolf Müller[4], Marcel A. Behr[1,2,3,5]

[1]Department of Microbiology & Immunology, McGill University, Montréal, Québec, Canada.
[2]Infectious Diseases and Immunity in Global Health Program, Research Institute of the McGill University Health Centre, Montréal, Québec, Canada.
[3]McGill International TB Centre, Montréal, Québec, Canada.
[4]Department of Microbial Natural Products, Helmholtz-Institute for Pharmaceutical Research Saarland (HIPS), Helmholtz Centre for Infection Research (HZI) and Department of Pharmacy, Saarland University, Campus E8 1, 66123 Saarbrücken, Germany
[5]Department of Medicine, McGill University Health Centre, Montréal, Québec, Canada.

Supporting Information

TABLE S1 Compounds with *in vitro* activity against *M. abscessus* identified from natural product library

Threshold Cut-off	Library	Compound	Source	Mechanism	Reference
10%	Bio	Actinomycin D	*Streptomyces* spp.	DNA intercalator	[1]
	Bio	Actinomycin X2	*Streptomyces* spp.	DNA intercalator	[1]
	Bio	Becatecarin	Derivative of rebeccamycin (*Nocardia* spp.)	DNA intercalator	[2]
	Bio	Echinomycin	*Streptomyces* spp.	DNA intercalator	[3]
	Bio	Telithromycin	Semisynthetic erythromycin derivative	50S ribosome inhibitor	[4]
	Myxo	Hyaboron	*Hyalangium minutum*	Putative potassium ionophore	[5]
	Myxo	Lysobactin (Katanosin B)	*Lysobacter* spp.	Lipid II inhibitor	[6]
	Myxo	Lysocin E	*Lysobacter* spp.	Lipid II/Menaquinone inhibitor	[7]
	Myxo	Lysocin I	*Lysobacter* spp.	Lipid II/Menaquinone inhibitor	[7]
	Myxo	Myxovalargin A	*Myxococcus fulvus*	Inhibitor of protein synthesis	[8]
	Myxo	Sorangicin A	*Sorangium cellulosum*	RNAP inhibitor	[9]
	Myxo	Neosorangicin A	*Sorangium cellulosum*	RNAP inhibitor	[10]
50%	Bio	Simocyclinone D4	*Streptomyces antibioticus* Tü 6040	DNA gyrase inhibitor	[11]
	Bio	Simocyclinone D8	*Streptomyces antibioticus* Tü 6040	DNA gyrase inhibitor	[11]
	Bio	Aranciamycin	*Streptomyces* spp.	Collagenase inhibitor	[12]
	Bio	Pyridomycin	*Dactylosporangium fulvum*	Enoyl-ACP reductase inhibitor	[13]
	Bio	Cochliodone A	*Chaetomium cochliodes*	N.D[a]	[14]
	Bio	Bafilomycin A1	*Streptomyces* spp.	Vacuolar-type H^+-ATPase inhibitor	[15]
	Myxo	Tartrolon B	*Sorangium cellulosum*	Disrupt K^+ gradient	[16]
	Myxo	Tartrolon C	*Streptomyces* spp.	N.D[a]	[17]

[a]Not determined

TABLE S2 Effect of carbon source and minimal media on potencies of natural product hits against *M. abscessus* ATCC 19977 smooth reference strain

	MIC_{90} (µM)			
**Compound**	**7H9[a] Glycerol**	**7H9[a] Acetate**	**CaMH[b] Glycerol**	**Sauton[c] Glycerol**
Lysobactin	3	3	4	5
Sorangicin A	13	16	16	39

[a]Middlebrook 7H9 media with 10% ADC (albumin, dextrose, catalase), 0.05% Tween-80, 0.02% carbon source
[b]Cation-adjusted Mueller Hinton media with 10% ADC, 0.05% Tween-80, 0.02% carbon source
[c]Sauton mycobacteria minimal media with 0.05% Tween-80, 0.02% carbon source

TABLE S3 Potencies of natural product hits against *M. abscessus* reference strain and clinical isolates

					MIC_{90} (µM)[a]				
					**Cell Wall**		**RNA Polymerase**		
**Isolate**	**Subspecies**	**Morphotype**	***erm*41 Sequevar**	**CLR susceptibility**	**LYB**	**VAN**	**SOR**	**RIF**	**RFB**
ATCC19977	abscessus	Smooth	T28	Sensitive	2	7	11	13	2
ATCC19977	abscessus	Rough	T28	Sensitive	2	9	18	23	2
MB084806	abscessus	Smooth	T28	Sensitive	5	2	7	7	1
MB092927	abscessus	Smooth	C28	Sensitive	8	3	21	18	2
MB093261	abscessus	Smooth	T28	Sensitive	9	7	17	10	2
L0007906	abscessus	Rough	T28	Resistant	13	5	15	69	5
MB086151	abscessus	Rough	C28	Sensitive	5	2	13	12	1
MB088425	massiliense	Smooth	deletion	Sensitive	4	6	8	7	2
MB088215	massiliense	Smooth	deletion	Sensitive	4	5	8	7	2
MB092961	massiliense	Rough	deletion	Sensitive	4	5	8	7	1
L00042522	massiliense	Rough	deletion	Resistant	19	8	50	100	7
AV	massiliense	Smooth	deletion	Sensitive	15	8	30	46	3
167P	bolletii	Rough	T28	Resistant	5	2	3	4	1

[a]CLR, clarithromycin; LYB, lysobactin; VAN, vancomycin; SOR, sorangicin A; RIF, rifampicin; RFB, rifabutin

TABLE S4 Drug susceptibility profile of *M. abscessus* complex clinical isolates.

			MIC$_{90}$ (μM)[ab]									
			Protein Synthesis					DNA Synthesis		ETC		Cell Wall
**Isolate**	**Subspecies**	**Morphotype**	**CLR**	**AMK**	**LZD**	**TIG[c]**	**DOX**	**MOX**	**CIP**	**BDQ[c]**	**CFZ[c]**	**CFX**
MB084806	abscessus	Smooth	1	11	*36*	4	**120**	*3*	**31**	0.1	6	15
MB092927	abscessus	Smooth	0.3	3	*93*	3	**116**	*6*	**74**	1	7	28
MB093261	abscessus	Smooth	1	8	**97**	5	**120**	*4*	**33**	1	9	18
L0007906	abscessus	Rough	**>134**	**>172**	10	9	**28**	*10*	**28**	3	11	30
MB086151	abscessus	Rough	0.3	6	10	4	**107**	*3*	**29**	1	44	*38*
MB088425	massiliense	Smooth	1	11	5	9	**58**	2	*8*	1	10	17
MB088215	massiliense	Smooth	1	11	5	5	**61**	*3*	*8*	1	10	16
MB092961	massiliense	Rough	1	11	5	5	**56**	2	*7*	2	11	7
L00042522	massiliense	Rough	**>134**	*42*	*72*	>68	*13*	**13**	**41**	2	25	15
MB087124	massiliense	Rough	**>134**	*37*	*53*	10	*13*	**13**	**17**	3	24	15

[a]CLR, clarithromycin; AMK, amikacin; LZD, linezolid; TIG, tigecycline; DOX, doxycycline; MOX, moxifloxacin; CIP, ciprofloxacin; BDQ, bedaquiline; CFZ, clofazimine; CFX, cefoxitin
[b]Susceptible, *Intermediate*, **Resistant**
[c]No established critical concentration cut off

References

1. Hollstein U. Actinomycin. Chemistry and mechanism of action. Chem Rev. 1974;74: 625–652. doi:10.1021/cr60292a002
2. Robey RW, Obrzut T, Shukla S, Polgar O, MacAlou S, Bahr JC, et al. Becatecarin (rebeccamycin analog, NSC 655649) is a transport substrate and induces expression of the ATP-binding cassette transporter, ABCG2, in lung carcinoma cells. Cancer Chemother Pharmacol. 2009;64: 575–583. doi:10.1007/s00280-008-0908-2
3. Kong D, Park EJ, Stephen AG, Calvani M, Cardellina JH, Monks A, et al. Echinomycin, a small-molecule inhibitor of hypoxia-inducible factor-1 DNA-binding activity. Cancer Res. 2005;65: 9047–9055. doi:10.1158/0008-5472.CAN-05-1235
4. Zhanel GG, Walters M, Noreddin A, Vercaigne LM, Wierzbowski A, Embil JM, et al. The Ketolides: A Critical Review. Drugs. 2002;62: 1771–1804. Available: https://link.springer.com/content/pdf/10.2165/00003495-200262120-00006.pdf
5. Surup F, Chauhan D, Niggemann J, Bartok E, Herrmann J, Keck M, et al. Activation of the NLRP3 Inflammasome by Hyaboron, a New Asymmetric Boron-Containing Macrodiolide from the Myxobacterium Hyalangium minutum. ACS Chem Biol. 2018;13: 2981–2988. doi:10.1021/acschembio.8b00659
6. Lee W, Schaefer K, Qiao Y, Srisuknimit V, Steinmetz H, Müller R, et al. The Mechanism of Action of Lysobactin. J Am Chem Soc. 2016;138: 100–103. doi:10.1021/jacs.5b11807
7. Santiago M, Lee W, Fayad AA, Coe KA, Rajagopal M, Do T, et al. Genome-wide mutant profiling predicts the mechanism of a Lipid II binding antibiotic article. Nat Chem Biol. 2018;14: 601–608. doi:10.1038/s41589-018-0041-4
8. Irschik H, Reichenbach H. The mechanism of action of myxovalargin a, a peptide antibiotic from myxococcus fulvus. J Antibiot (Tokyo). 1985;38: 1237–1245. doi:10.7164/antibiotics.38.1237
9. Lilic M, Chen J, Boyaci H, Braffman N, Hubin EA, Herrmann J, et al. The antibiotic sorangicin A inhibits promoter DNA unwinding in a Mycobacterium tuberculosis rifampicin-resistant RNA polymerase. Proc Natl Acad Sci U S A. 2020;117: 30423–30432. doi:10.1073/pnas.2013706117
10. Müller R, Zaburannyi N, Herrmann J, Jansen R, Mohr K, Karwehl S. Novel Sorangicin Antibiotic. EP3498714A1, 2020.
11. Flatman RH, Howells AJ, Heide L, Fiedler HP, Maxwell A. Simocyclinone D8, an inhibitor of DNA gyrase with a novel mode of action. Antimicrob Agents Chemother. 2005;49: 1093–1100. doi:10.1128/AAC.49.3.1093-1100.2005
12. Bols M, Binderup L, Hansen J, Rasmussen P. Inhibition of Collagenase by Aranciamycin and Aranciamycin Derivatives. J Med Chem. 1992;35: 2768–2771. doi:10.1021/jm00093a008
13. Hartkoorn RC, Sala C, Neres J, Pojer F, Magnet S, Mukherjee R, et al. Towards a new tuberculosis drug: Pyridomycin - nature's isoniazid. EMBO Mol Med. 2012;4: 1032–1042. doi:10.1002/emmm.201201689
14. Phonkerd N, Kanokmedhakul S, Kanokmedhakul K, Soytong K, Prabpai S, Kongsearee P. Bis-spiro-azaphilones and azaphilones from the fungi Chaetomium cochliodes VTh01 and C. cochliodes CTh05. Tetrahedron. 2008;64: 9636–9645. doi:10.1016/j.tet.2008.07.040
15. Wang R, Wang J, Hassan A, Lee CH, Xie XS, Li X. Molecular basis of V-ATPase inhibition by bafilomycin A1. Nat Commun. 2021;12. doi:10.1038/s41467-021-22111-5
16. Schummer D, HöFle G, Reichenbach H. The Tartrolons, New Boron-containing Antibiotics from a Myxobacterium, Sorangium cellulosum. J Antibiot (Tokyo). 1995;48: 26–30. doi:10.7164/antibiotics.48.26
17. Lewer P, Chapin EL, Graupner PR, Gilbert JR, Peacock C. Tartrolone C: A novel insecticidal macrodiolide produced by Streptomyces sp. CP1130. J Nat Prod. 2003;66: 143–145. doi:10.1021/np020451s
